# Perceived neighbourhood affluence, mental health and wellbeing influence judgements of threat and trust on our streets: An urban walking study

**DOI:** 10.1371/journal.pone.0202412

**Published:** 2018-08-16

**Authors:** Rhiannon Corcoran, Rosie Mansfield, Christophe de Bezenac, Ellen Anderson, Katie Overbury, Graham Marshall

**Affiliations:** 1 Institute of Psychology Health and Society, University of Liverpool, Liverpool, United Kingdom; 2 School of Education, University of Manchester, Greater Manchester, United Kingdom; 3 Greater Manchester Mental Health Trust, Greater Manchester, United Kingdom; 4 Director, Prosocial Place, Liverpool, United Kingdom; Rice University, UNITED STATES

## Abstract

This study aimed to understand how people respond to different urban neighbourhoods. We explored whether participants’ mental health and wellbeing, judgements of resident wealth, family SES and sentiments reflected in descriptions of place features predicted in situ sense of threat and trust. Forty-six student participants walked in groups through 2 urban neighbourhoods, separated by a park, in the North West of England, noting responses at pre-determined stops. Significant differences existed in participants’ sense of trust and threat between the 2 neighbourhoods along with differences in perceived resident wealth and sentiments expressed. Participants’ levels of persecutory ideas and their sense of residents’ wealth predicted in situ trust in both neighbourhoods while level of personal resilience predicted the extent of threat felt in the more deprived neighbourhood. Demonstrating the value of the method, these findings have implications for the governance of urban neighbourhoods whereby obvious cues signalling a harsh environment need to be minimised to create more positive psychological responses to places.

## Introduction

### The experienced city and the urbanicity effect

The ‘perceived’ city is an experiential tapestry woven from physical characteristics, uses and abuses of space, a sense of the resident community and many more abstract features combining to elicit context-sensitive thoughts, feelings and sensations. These psychological responses enable us to use rules of thumb to make speedy judgments about the places we visit or pass through. The role of individual mood and in situ emotional reactions in the making of these heuristic judgements is likely to be critical, as it is these adaptive responses to place that inform our basic survival choices to fight, flee or dwell. The understanding of place as a complex socio-emotional experience is captured by Cresswell’s [1] definition of place as ‘space endowed with meaning’. It is epitomised by the ‘urbanicity effect’, the well-established finding that both common and severe mental health conditions are more prevalent in inner cities than elsewhere [2–5]. However, while the causes of the urbanicity effect remain contested [6–12], there has been very little psychological research into its foundations.

### Urban deprivation and mental distress

Urban deprivation is a significant public health concern and a factor that undeniably accounts for some of the urbanicity effect. The UK’s Department of Communities and Local Government [13] acknowledged the high concentration of deprived areas in inner cities of the UK. However, the urbanicity effect has been found to exist even after statistically controlling for deprivation suggesting that other factors, including psychological responses to places, are part of the picture [11].There is also evidence to suggest a causal relationship between perceived social characteristics of place and risk of mental health difficulties [7, 14, 15] with most authors endorsing a cyclic relationship between the psychology of mental distress and the urban environment with place influencing people’s psychology and vice versa [16–19]. Taken together, these studies emphasise the inherently contextual nature of psychological responses to living environments and the close ties that exist between place characteristics and human wellbeing.

Depression, anxiety and paranoid ideation all share the tendency to anticipate and avoid social threat. These mental health related tendencies include a heightened attention to threat along with an over-estimation of future threatening events evident in both clinical and non-clinical samples [20–24]. Those who live in disordered and deprived urban neighbourhoods also report increased perception of threat and hostility [18]. In a 2014 study by Hill, Pollet and Nettle [25],that emphasised the relationship between heightened attention to threat and perceived neighbourhood disorder, the dynamic, context-dependent nature of individuals’ responses to living environments was articulated.

As well as threat, the physical characteristics of places have been shown to effect levels of trust, a variable that correlates strongly with social capital [26]. For example, the walkability of a neighbourhood can predict levels of trust with mixed-use neighbourhoods showing higher levels of social capital [27]. It is believed that physical, socio-economic and environmental cues, as well as in-group/out-group assumptions drive changes in trust-related behaviours such that perceived trustworthiness within a neighbourhood has been found to be positively associated with similar socio-economic status and negatively associated with ethnic heterogeneity [28–30].

The fact that our psychological responses are so closely bound up with the contexts in which we find ourselves is summarised in the so-called ‘broken window theory’ where it is argued that inferences about both trust and threat are drawn on the basis of the physical and social environment. This theory is supported by findings such as those referred to above and one that shows how the amount of litter in an area is directly correlated with perceptions of the likelihood of theft [31].

### Brief exposure to harsh environments

It seems that even passing exposure to harsh urban environments can have measurable negative psychological effects in both clinical and nonclinical groups. The Camberwell walking study [32] explored the acute psychological effects of exposure to a relatively harsh urban environment on people experiencing persecutory delusions. These authors found increased feelings of anxiety, persecution and negative beliefs about others following a brief walk through the local inner city neighbourhood. In a study exploring the effects of dwelling as well as passing through, Nettle, Pepper, Jobling, & Schroeder [33] compared the level of social trust and paranoia of residents of two contrasting neighbourhoods. Residents in the deprived neighbourhood displayed significantly lower levels of social trust and significantly higher levels of paranoia compared to the residents of the affluent neighbourhood. Interestingly, student visitors who had spent just 45 minutes delivering questionnaires to houses in either one of the two unfamiliar neighbourhoods, reported almost the same magnitude of difference in levels of trust and paranoia as reported in the residents. By showing that acute measurable differences in social trust and paranoia, following exposure to a harsh living environment mimics the psychological profile of residents of the area, Nettle et al.’s study suggests that these responses to harsh living environments could be universal. This proposition is supported by the fact that the demographic, socioeconomic background of the residents and the student visitors were likely to have been quite different. We contend that universal emotional responses to harsh environments, measurable after brief exposure but enduring within residents, become a chronic source of inescapable stress underpinning the urbanicity effect. However, Nettle et al.’s study has 3 important limitations. First, the two urban neighbourhoods to which the students were briefly exposed were not matched for urban morphology. Second, as different students went to each area, individual differences in trait level differences between the student groups in variables such as such as anxiety, personal resilience and socio-economic status may have played a part in determining the findings. Finally, no information was collected about what specifically the students were responding to in the environments.

Just as, the between subjects design used in [33] meant that the interaction between individual differences and distinct areas could not be explored, other previous in situ studies have used between subjects designs and, furthermore, they have failed to use appropriate comparison tasks. For example, Ellet et al. [32] used a mindfulness control task instead of comparing responses to a more affluent urban neighbourhood. The authors acknowledged that future research was needed to identify features of the environment that may drive changes in psychological processes.

Such experimental limitations need to be addressed if urban psychological research and methods is to be used to improve the impact of urban living environments on human wellbeing.

### Aims and hypotheses of the current study

With a particular focus on in situ sense of threat and trust, the current study used an experience-sampling booklet to gain quantitative and qualitative data at consecutive place nodes along a route passing through 2 distinct urban neighbourhoods differing in level of deprivation and in ethnic composition. The neighbourhoods were well controlled in terms of urban morphology, presence of heritage buildings, a main street, number of shops, restaurants, houses, roads and green spaces. The collection of quantitative and qualitative information provided by the same individuals walking through two neighbourhoods aimed to reveal comparative subjective assessments between the neighbourhoods that could take into account any effects of individual differences in the participants. In particular, this study aimed to explore how the participants’ pre-existing levels of non-clinical depression, anxiety, persecutory feelings and personal resilience influence the in situ judgments they make about the contrasting urban environments.

It was hypothesised that:

- In situ inferences about threat and trust would differ significantly between the 2 neighbourhoods along the walking route.
- Reflecting the importance of cues to deprivation as well previous research emphasising the importance of similar socio-economic status [28,29], it was hypothesised that in situ sense of threat and of trust would be predicted by the sense of wealth in the neighbourhood.
- The walkers’ subjective judgement about their family’s social-economic status would predict their in situ judgments of threat and trust. In particular, with these student walkers and reflecting prior research [28,29], family SES would correlate positively with trust and negatively with threat in the better off area while correlating negatively with trust and positively with threat in the more deprived area.
- The neighbourhood features that the walkers described would differ in terms of sentiment represented, measured by sentiment analysis software. Specifically, we expected higher levels of negative sentiment to be expressed in the more deprived neighbourhood. Further, and in line with the assumption that what we perceive influences the judgement we make, we expected that the sentiment expressed would be related to judgements of threat and trust.
- In accordance with the urbanicity effect and the findings of previous research on the effects of brief exposure to harsh environments [32, 33], baseline measures of mental health and wellbeing were expected to predict in situ threat and trust in the neighbourhoods with stronger correlations existing in the more deprived neighbourhood.

## Materials and methods

### Ethical statement

The research was approved by the authors' Institutional Review Board, University of Liverpool Research Ethics Committee Reference number IPHS-1213-LB-114. Before setting off on the walk or completing any baseline measures the participants were either sent by email or given an information sheet about the study and invited to ask the research team if they had any questions in a process agreed through the University Research Ethics Committee. Informed written consent was collected from all participants using a study consent form. On the days of the scheduled walks, participants were reminded that they were free to withdraw from the study at any time.

### Selecting the route

**Fig 1:**
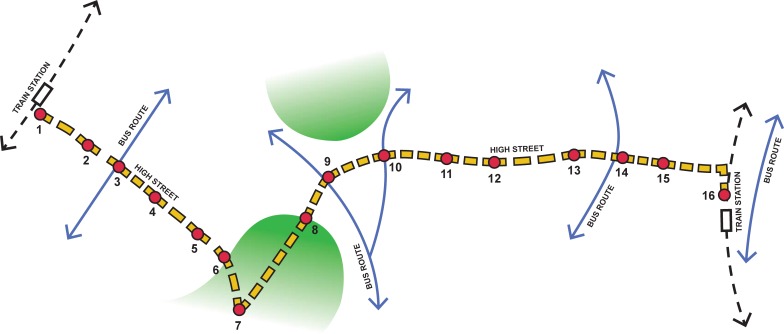
Diagram of the walk, stops 1–16 neighbourhood 1 = stops 1–6. Neighbourhood 2 = Stops 10–1.

The chosen route of approximately 2 miles lay between two train stations and embedded two distinct urban neighbourhoods that were separated by a large park. Using a Lynchian approach [34], an urban design professional selected 16 consecutive ‘place nodes’ where the walk would halt to enable data collection. Lynch, an urban planner, suggested that certain core characteristics (i.e. identity, structure, meaning transparency and congruence) help to define place. These characteristics are functionally linked to certain forms, structures and levels of organization—paths, edges, nodes, landmarks and districts—that together make places more legible by aiding the formation of a mental map. We aimed to make the stops along the walking route conform to Lynchian concepts of place while also aiming for their equidistance along the route. For analysis purposes, the route was divided into three parts; two contrasting urban neighbourhoods made up each end of the walk (stops 1–6, Neighbourhood 1 and stops 10–15 Neighbourhood 2). They were separated by the park (stops 6–9). As green spaces are believed to have ‘restorative’ qualities, able to attenuate negative emotions and buffer against stress [35], walking into and out of the park was used to ‘wash out’ the psychological effects of the contrasting urban neighbourhoods. The main purpose of the section of walk through this heritage parkland was to act as a zone of separation between the two neighbourhoods so that we could be more certain that recordings taken at the first few stops within each neighbourhood would reflect that neighbourhood rather than reflecting a remnant reaction to the previous neighbourhood. Both neighbourhoods were dense 19^th^ century areas, 2–3 miles from the city centre and similarly comprised of a busy main street with shops, restaurants and similar architecture, roads, green areas with adjacent traditional as well as more contemporary residential development of similar quality. Importantly, analysis of neighbourhood statistics showed large contrasts between the two neighbourhoods Fig 1. is a diagram of the walk and Table 1 provides relevant neighbourhood statistics.

**Table 1:** Summary statistics for neighbourhoods 1 and 2.

Indicator	Neighbourhood 1	Neighbourhood 2	The City Average
1. IMD—% of Area in Most Deprived 10% Nationally	22.1	86.6	49.6
2. Average Household Income	£35,506	£23,066	£29,379
3. Anti-Social Behaviour per 1,000 Persons	42.6	56.7	51.2
4. % Ethnic minority residents	15.5	45.5	13.7

1. Department of Communities and Local Government (2010)

2. CACI Paycheck (2014)

3. City Council CitysafeTeam (2013/14) behaviours included as anti-social include reports to the authorities of general vandalism and graffiti; drinking alcohol or purchasing drugs on the street; threatening, verbally abusive or drunken behaviour; rowdy neighbours; abandoned vehicles; use of off-road motorbikes on roads; setting off fireworks late at night; fly tipping or rubbish dumping; littering; uncontrolled dogs/ pets. This information is provided at https://www.merseyside.police.uk/advice-and-protection/anti-social-behaviour/.

4. Census (2011) % of ethnic minority residents by ward

Walks took place in 2014, the statistics are the most recent available at the time of the study

### Participants

An equal number of male and female university students provided data for this study (males *N* = 23, females *N* = 23). 44/46 participants were aged 18–24 with 32/46 classifying themselves as White/White British. 39/46 participants regarded their family socio-economic status to be above average compared to the UK population. For pragmatic reasons of data collection, participants walked in groups of 7–8 in 2 x mixed groups (3–4 male, 4 female), 2 x all male groups (8) and 2 x all female groups (7–8). In the majority, the members of the walking groups were strangers. They were asked not to confer with others as they recorded their responses at the stops along the walk. To be confident that the responses reflected the individual’s own judgement, the research team gently reminded participants of the need to abide by this rule if the need arose.

### Measures

#### Baseline measures

Participants provided information about their age, sex, ethnicity, symptoms of mental distress and subjective socioeconomic status.

As severe mental distress such as schizophrenia is characterised by paranoid or persecutory ideas and because schizophrenia has consistently been found to be more prevalent in inner cities, we included a measure of this type of thinking as seen in non-clinical samples, supporting a continuum model of persecutory ideation. The purpose was to explore how pre-existing levels of persecutory beliefs affected in situ trust and threat responses in urban living environments. To measure this tendency we used the Persecution Scale from the Persecution and Deservedness Scale (PaDS) [36]: This 10-item measure of paranoid ideation is suitable for use with both non-clinical and clinical samples. Statements refer to direct or indirect intentional harm from others and respondents are asked to rate the extent to which they agree with each statement (0 = certainly false; 4 = certainly true). The authors of the scale report high internal consistency in a non-clinical, student sample (α = 0.84).

The 21-item self-report Depression, Anxiety and Stress Scale (DASS 21) [37]: is used to measure the severity of symptoms relating to depression, anxiety and stress. Participants rate the extent to which items applied to them on a 4-point Likert scale (0 = did not apply to me at all; 3 = applied to me very much, or most of the time). When compared with other measures of depression and anxiety, DASS21 has been found to have high internal consistency (α = 0.93; CI_95_ = 0.93 to0.94), and good convergent and discriminant validity in a non-clinical adult sample.

As we were interested to find out if a general tendency towards personal resilience (i.e. being able to bounce back following experiences of adversity) can protect from the acute effects of exposure to harsh environments. We used the Brief Resilience scale [38] to measure this tendency so that we could include it as a potentially protective individual difference variable of mental health and wellbeing. The authors report excellent internal consistency ranging between .80-.91 in their studies using this 6 item scale with good convergent validity and test-retest reliability also demonstrated.

The MacArthur Scale of Subjective Social Status (SES) [39] is a subjective measure of SES presented visually using a 10- rung ladder. The top rung (10) indicates a perception of being a lot better off, in terms of money, education and employment than the UK population and the bottom rung (1) indicates a perception of being a lot worse off. The original MacArthur scale was adapted to gain student’s perceived family SES (UK).

#### Repeated measures

Walkers were asked to complete the following measures in the experimental booklet at each stop along the walk.

In situ judgements of sense of threat, trust and wealth: Participants were asked how likely they felt it was that a potentially threatening event would occur in the next five minutes (0 = I am completely sure nothing potentially threatening is going to happen in the next 5 minutes—100 = I am completely sure something potentially threatening is going to happen in the next 5 minutes)

Participants were asked to indicate how trustworthy they believed the residents of the area to be with 0 indicating that the residents were considered to be totally untrustworthy and 100 indicating that the residents were considered to be totally trustworthy. Participants were also asked to indicate how rich or poor they believed the residents of the area to be with 0 indicating that residents were considered to be extremely poor, and 100 indicating that residents were considered to be extremely rich.

At each stop participants were asked to write down a brief description of what had ‘caught their eye’ or what they regarded to be the main feature in the vicinity of the stopping place. These descriptions were collated for the 6 stops in each of the 2 neighbourhoods, averaged and subjected to a sentiment/emotion analysis using the R [https://cran.r-project.org/] package Cognizer [https://github.com/ColumbusCollaboratory/cognizer#emotion] to wrap function calls to IBM Watson services [https://www.ibm.com/watson/products-services/]. In order to quantify the extent of positive/negative overall sentiment reflected as well as to evaluate the statements for specific sentiments expressed. In particular, the sentiments of anger, disgust, fear, sadness and joy were calculated and examined. The software also supported the production of word clouds that depict a summarised content of the written descriptions by emotional category.

#### Immediate post-walk measures

As the weather on the day was likely to influence mood, at the end we asked the walkers to rate the weather during the walk using a 10-point Likert scale (1 = extremely bad weather i.e. heavy rain and wind and 10 = extremely good weather i.e. a perfectly clear sunny day). At the final stop, participants reported any areas along the walk that were familiar to them. These familiarity statements revealed that 31/46 walkers were largely unfamiliar with the neighbourhoods in question and 15 had some minimal knowledge of one or both of the neighbourhoods.

### Procedure

Prior to their walk, all participants completed a survey to collect baseline measures of mental health. For all participants these measures were collected at least 1 week before the date of the group walk they would attend. An information sheet and instructions about getting to the starting point of the walk was emailed to all participants. The beginning of the walk was counterbalanced, with half of the walkers starting at the station in neighbourhood 1 (*N* = 23) and half from the station in neighbourhood 2 (*N* = 23). All walks set off at approximately 1.30 pm. Before departure, the use of the experimental booklet was explained.

## Results

### Neighbourhood differences in judgements of threat, trust and wealth

Table 2 provides summary statistics of the walkers’ summed judgements of in situ sense of threat, trust and wealth across the 6 stops within each of the 2 neighbourhoods. Paired t-tests showed that both sense of threat and trust differed significantly between neighbourhoods with neighbourhood 2 significantly higher in sense of threat and lower in sense of trust than neighbourhood 1 (Paired t-test (threat) = -5.216, 45df, p<0.001, 2 tailed test; Paired t-test (trust) = 8.163, 45df, p<0.0001, 2 tailed test). The neighbourhoods also differed significantly in terms of how wealthy the walkers judged their residents to be, with people dwelling in neighbourhood 1 considered to be significantly wealthier than those dwelling in neighbourhood 2 (Paired t- test = 9.759; 45df, p < .0001, 2 tailed test).

**Table 2:** Descriptive statistics (mean & (sd)) for in situ neighbourhood judgements of threat, trust, wealth and sentiment analysis scores.

	Neighbourhood 1	Neighbourhood 2	Paired t test p. level 2 tailed
Sum of in situ threat judgements	82.61 (73.2)	148.48 (106.96)	P <0.0001
Sum of in situ trust judgements	345.35 (122.9)	243.33 (136.64)	P<0.0001
Sum of in situ wealth judgements	354.59 (78.77)	252.06 (69.47)	P<0.0001
Average Overall valence	0.17 (0.17)	0.03 (0.169)	P<0.0001
Average Anger	0.08 (0.047)	0.11 (0.062)	P<0.001
Average Disgust	0.07 (0.051)	0.09 (0.059)	P = 0.05
Average Fear	0.10 (0.052)	0.13 (0.064)	P<0.001
Average Sadness	0.12 (0.084)	0.15 (0.076)	Ns
Average Joy	0.27 (0.133)	0.23 (0.102)	Ns

### Correlations between judgements about neighbourhood threat and trust, wealth, weather and family SES

The bivariate Pearson’s correlations between neighbourhood judgements of trust and threat and weather, family SES and judgement of neighbourhood wealth are shown in Table 3.The number of relationships found differed between the neighbourhoods. Sense of trust correlated with sense of wealth and the weather rating in both neighbourhoods while in neighbourhood 2 trust also correlated with family SES. Sense of threat correlated with family SES, and with the sentiments of disgust and joy but only in neighbourhood 2.

**Table 3:** Pearson’s correlations for in situ neighbourhood judgements of threat and trust with judgements of resident wealth, assessment of weather during the walk, subjective family SES and sentiment expressed in descriptions.

	Neighbourhood 1	Neighbourhood 2
Threat x Trust	-0.04	-0.145
Threat x Wealth	-0.166	-0.116
Threat x Weather	-0.213	-0.069
Threat x Family SES	0.169	0.277 (p<0.05, 1 tailed)
Threat x Overall Sentiment	-0.166	-0.240
Threat x Anger	0.07	-0.131
Threat x Disgust	-0.142	0.295 (p<0.05, 1 tailed)
Threat x Fear	-0.10	0.080
Threat x Sadness	-0.035	0.109
Threat x Joy	0.014	-0.217 (p<0.05, 1 tailed)
Trust x Wealth	0.407 (p<0.005, 1 tailed)	0.527 (p<0.005, 1 tailed)
Trust x Weather	0.272 (p<0.05, 1 tailed)	0.347 (p<0.05, 1 tailed)
Trust x Family SES	-0.138	-0.247 (p<0.05, 1 tailed)
Trust x Overall Sentiment	0.199	0.217
Trust x Anger	0.160	0.049
Trust x Disgust	-0.006	-0.132
Trust x Fear	-0.003	-0.027
Trust x Sadness	-0.129	-0.065
Trust x Joy	0.104	0.227

### Neighbourhood sentiment analysis: Differences and correlations

Table 2 provides the descriptive quantitative data obtained from the sentiment analyses carried out on the short descriptions provided by the walkers at the stops within the neighbourhoods. The overall sentiment score for both neighbourhoods was marginally positive such that the walkers’ descriptions reflected slightly more positive than negative sentiment. However, the extent of positivity was significantly higher for neighbourhood 1 than it was for neighbourhood 2, (Paired t-test (overall sentiment) = 4.917, 45 df, p<0.001, 2 tailed). In terms of the specific sentiments explored, expressions of anger and fear differed significantly between the neighbourhoods with higher levels of both found within the descriptions of neighbourhood 2 (t (anger) = -2.80. 45 df, p<0.01, 2 tailed; t(fear) = -2.804, 45df, p<0.01, 2 tailed). Expressions of disgust just failed to reach significance applying a conservative 2 tailed test (t (disgust) -2.017, 45 df, p = 0.05) with the same tendency for more of this negative affect expressed for the features of neighbourhood 2 than for those of neighbourhood 1. Neither expressions of sadness nor joy differed significantly between neighbourhoods.

The correlations between the expressed sentiments of neighbourhood features and neighbourhood threat and trust can be seen in See Table 3. We found significant correlations between sense of threat in neighbourhood 2 and the level of disgust and joy expressed in the walkers’ descriptions.

**Fig 2:**
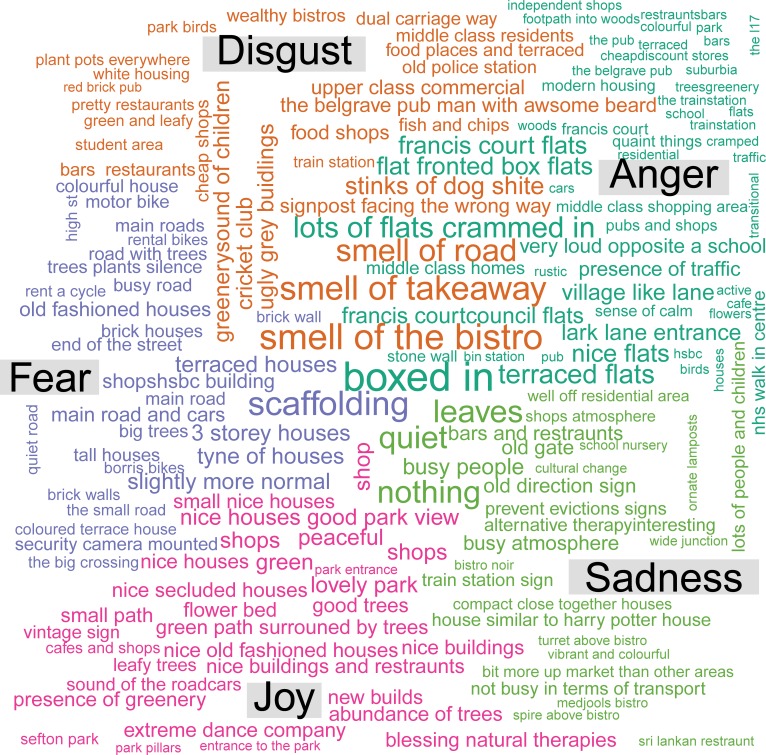
Word cloud of sentiments expressed in neighbourhood 1.

**Fig 3:**
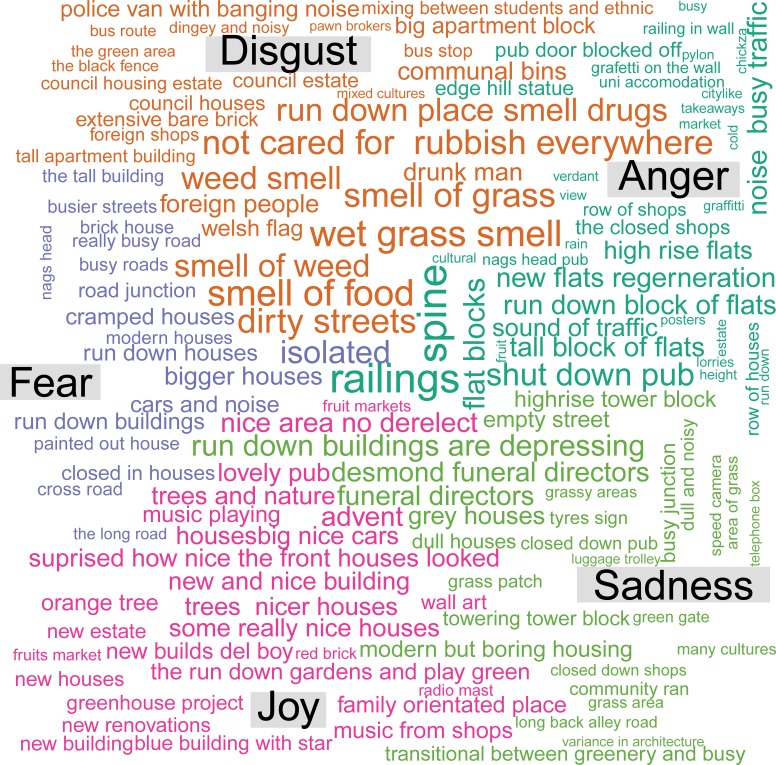
Word cloud of sentiments expressed in neighbourhood 2.

**Fig 4:**
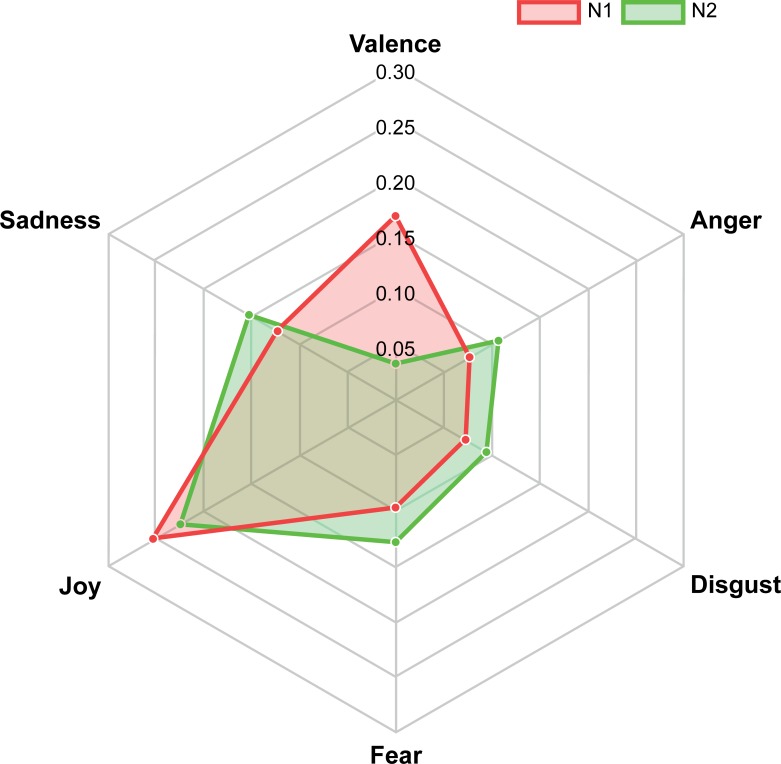
Sentiment analysis comparison of neighbourhoods 1 (red) and 2 (green).

The sentiments expressed within each neighbourhoods are shown in word clouds and in a diagrammatic contrast in Figs 2–4.

### Correlations between baseline mental distress and judgements of neighbourhood threat and trust

Table 4 provides the summary statistics relating to the mental health and wellbeing measures collected from all participants before the walk. Also shown in Table 4 are Pearson’s correlations between judgements of neighbourhood threat and trust and these mental health and wellbeing measures. While no significant correlations existed between DASS21 depression, anxiety and stress score and neighbourhood threat and trust, meaningful significant correlations did exist between the persecutory beliefs score, threat in neighbourhood 2 and trust in both neighbourhoods. A significant negative correlation between judgement of threat in neighbourhood 2 and personal resilience score was also found.

**Table 4:** Descriptive statistics (mean & (sd)) for mental health and wellbeing measures and their correlations with in situ neighbourhood judgements of threat and trust.

	Depression, Anxiety and Stress (DASS)	Paranoid feelings (PaDS)	Personal resilience (Brief Resilience Scale)
Mean and (sd)	13.93 (10.62)	12.74 (8.82)	20.67 (4.40)
X Threat Neighbourhood 1	0.196	0.214	-0.241
X Threat Neighbourhood 2	0.188	0.243 (p<0.05)	-0.376 (p<0.01)
X Trust Neighbourhood 1	-0.218	-0.390 (p<0.001)	-0.019
X Trust Neighbourhood 2	-0.158	-0.342 (p<0.01)	0.069

### Multiple regressions: Neighbourhood threat and trust

Finally, linear regressions using the enter method examined which of the significant bivariate correlates identified by the earlier analyses made significant independent contributions to the variance of in situ judgements of neighbourhood threat and trust.

As judgement of threat in neighbourhood 1 did not correlate significantly with any of the variables considered, no linear regression for this variable was undertaken.

With judgement of threat in neighbourhood 2 as the dependent variable, subjective family SES, sentiments of disgust and joy, persecutory beliefs score and personal resilience score were entered as independent variables into the regression equation. The model was significant (R = 0.582; Rsq. = 0.279; F = 3.096, p = 0.019). Only the personal resilience score of walkers made an independent contribution to the equation such that as self-reported personal resilience increased, judgements of threat in neighbourhood 2 decreased (beta = -0.361, t = -2.195, p = 0.034).

With judgement of trust in neighbourhood 1 as the dependent variable, sense of residents’ wealth, weather on the walk, and persecutory beliefs score were entered as independent variables into the regression equation. The model was significant (R = 0.583; Rsq. = 0.340; F = 7.223; p = 0.001) with significant independent contributions provided by judgements of residents’ wealth such that trust increased as wealth was judged higher (beta = 0.401, t = 3.166, p = 0.003) and the walkers’ persecutory beliefs scores with judgement of trust lower when persecutory beliefs were higher (beta = -0.369, t = -2.833, p = 0.007).

With judgement of trust in neighbourhood 2 as the dependent variable, sense of resident’s wealth, weather on the walk, subjective family SES and persecutory belief scores were entered as independent variables into the regression equation. The model was found to be highly significant (R = 0.714; Rsq.. = 0.510; F = 10.661; p = 0.000). Both residents’ wealth (Beta = 0.567. t = 5.00. p = 0.000) and persecutory beliefs (beta = -0.371, t = -3.096, p = 0.004) made significant independent contributions to the model such that lower judged resident wealth and higher walker persecutory beliefs predicted lower trust scores in neighbourhood 2.

## Discussion

### Summary of findings and their relation to the background literature

Using an ambitious mobile design, we collected data on walkers’ in situ judgements of threat and trust in 2 distinct urban neighbourhoods which differed in terms of deprivation. In summary, we found that while modelling sense of threat in the less deprived area was not statistically possible, sense of threat experienced in the more deprived neighbourhood was predicted by the walkers’ personal resilience scores collected before the walk. In situ sense of trust in both neighbourhoods was strongly predicted by the walkers’ judgements of the residents’ wealth and also by the walkers’ own persecutory belief scores collected prior to the walk. Certain negative sentiments reflected in the walkers’ descriptions of neighbourhood features characterised the more deprived neighbourhood with anger and fear most marked in contrast to the less deprived neighbourhood but, contrary to predictions, these sentiments were not retained as independent contributors to sense of threat or trust in the regression models.

The study has advanced the previous research that has used mobile methods to collect information on the psychological effect of urban areas [32, 33] by using a repeated measures design across a walk that embedded neighbourhoods contrasting in deprivation but which were otherwise very similar in terms of their main physical features. The use of the urban park as a restorative ‘wash out’ between the neighbourhoods, aimed to limit cross-neighbourhood contamination, was an important feature of this within participant design as was the counter-balancing of walk direction because fatigue and order effects such as increasing familiarity with the context over time will no doubt have affected the results in ways that are difficult to measure if not controlled for methodologically. The fact that this study also considered the effect of the walkers’ subjective family SES and the weather conditions on the walk is a further advance. Both of these variables, not considered in previous research, correlated significantly with in situ judgement of threat and trust at some level in the bivariate analyses. However, neither were found to contribute independently to the variance of either sense of trust or threat in the neighbourhood regression analyses.

### Appraisal of findings

As hypothesised, and consistent with previous findings [18, 25], the urban neighbourhoods that our walkers’ considered differed in terms of in situ judgements of threat and trust. The small, non-significant correlation between in situ judgement of threat and trust in both neighbourhoods was surprising as previous research endorsing the ‘broken window theory’ suggested that these variables, both elements of area social capital [26, 31] would share more variance than they did. That they did not might be accounted for by the different nature of the questions posed to elicit the walkers’ judgements. The threat question focused on anticipation of a threatening event occurring within minutes of the judgement being made. As such, it was a very stringent measure of sense of threat that resulted in smaller total scores across the 6 stops of each neighbourhood compared to the in situ trust judgements. This, as with the residents’ wealth judgement, was phrased more to elicit a general sense of the character of the residents. The similarity between the phrasing of the trust and wealth judgements may also go some way to explaining their high correlations in this data.

Consistent with published deprivation statistics [13], the 2 neighbourhoods also differed in terms of how wealthy their residents were assumed to be suggesting that certain cues in the environment enabled the walker to accurately assess these objectively measured differences. While we hypothesised that the assessment of residents’ wealth would correlate significantly with judgements of neighbourhood threat and trust, we had not anticipated the strength of the correlation between trust and wealth particularly. In the regression analyses of in situ trust in both neighbourhoods, perceived resident wealth accounted for the most variance by far. By contrast, it was not a significant predictor of sense of neighbourhood threat. That we tend to trust those who are judged to be relatively wealthy and that, according to this data, we do so independently of any sense of similarity in terms of family SES is a novel finding that warrants further research and, importantly, needs to be replicated using more diverse samples in different contexts.

Our findings in relation to measures of mental health and wellbeing are consistent with those of Nettle et al. [33] in that baseline measures of persecutory beliefs are related to judgements of trust in the neighbourhoods. These feelings, independent of depression, anxiety and stress are implicated in how we respond to unknown urban neighbourhoods in both of these brief exposure studies, indicating that the relationship is robust and may inform as to the nature of the urbanicity effect. It is notable that of the mental health conditions related to urban living, schizophrenia, that typically features feelings of paranoia, has the strongest ‘urbanicity effect’ while that for depression is weaker [2– 5]. However, the lack of relationship in this data between persecutory beliefs, depression, anxiety and sense of threat, contrasts with previous studies [20– 24]. It is likely that this disparity has something to do with the methods used to assess threat focus across the different studies and may relate to the precise wording of the in situ threat anticipation question used here which is a measure of state and not trait threat.

Our study has demonstrated the feasibility of using sentiment analyses to assess people’s implicit emotional reactions to place through their brief descriptions of salient features. This objective analysis of the content of written place feature descriptions was able to capture what was, before now, no more than a strong intuitive hunch that urban neighbourhoods are characterised by sensory cues (often but not always, visual) that illuminate their level of deprivation and that elicit meaningful, typically negative, emotions in those who notice them. While the overall valence expressed in both neighbourhoods was marginally more positive than negative is important, this marginal positivity was significantly higher in the less deprived neighbourhood and this, alongside the finding that sentiments of anger and fear were more marked in the deprived neighbourhood, speaks strongly to the need to address inequalities in these sensory cues to reduce negative emotional responses to places. Of course, before being able to address this effectively, there is a need to understand better what these cues are and how and when our attention is drawn to them and for how long they affect us. Future research making use of unobtrusive physiological monitoring with mobile eye tracking could usefully provide such information.

### Study limitations

While the several strengths of our study have undoubtedly advanced the way in which psychological responses to urban environments are studied, it is equally true that it suffers methodological limitations that have implications for how generalizable its findings are. The first and most important of these is the sample of walkers. Although one of our aims was to explore the utility of this method for the collection of rich, layered data, it is very likely that, with the possible exception of the relationships between place judgements and mental health and wellbeing, we would have found different patterns in the data if the walkers had come from different socio-economic and ethnic backgrounds. The majority of the student walkers subjectively placed their families as above average on socio-economic status and, although both neighbourhoods were deprived compared to the national average, it is likely that neighbourhood 1 would have felt more like ‘home’ to the student walkers than neighbourhood 2. With previous research highlighting the importance of the role of ‘alikeness’, similar SES and ethnicity in determining social capital judgements [28–30], the differences between those who walked through the neighbourhoods and those who reside in them will have played a critical role in determining the findings of the study and would have been more apparent for neighbourhood 2 than neighbourhood1. It seems likely that walkers who placed themselves as average or below for UK SES would have felt different things and perhaps judged threat and trust differently in the distinct neighbourhoods, emphasising the importance of individual differences in life circumstances. Future research should aim to use this method with more diverse groups of participants, including those with clinical histories and those who identify as ethnic minorities. This will help us to understand what if any psychological responses to objectively deprived and disorder neighbourhoods can be considered universal and thus candidate mechanisms underpinning the urbanicity effect. Such research would be complemented by social science studies aimed at understanding and addressing the judgement biases associated with the social characteristics of unfamiliar, deprived neighbourhoods that visitors from contrasting areas implicitly apply. Such automatic assumptions undoubtedly play a part in sustaining negative place perceptions and place stigma.

The importance of the difference in ethnic mix between the neighbourhoods of this study, where neighbourhood 2 was a more diverse and established ethnically-rich community is likely to have influenced the judgements that our student walkers made [30]. While we did not consider this directly in the analyses, we acknowledge its existence and recognise that ethnic heterogeneity is characteristic of many deprived urban areas in the UK. The co-ethnicity of the participants with the predominant ethnicity of the residents in neighbourhood 1 but not neighbourhood 2 can be considered a limitation of this work and something that needs addressing in future research.

For practical reasons to do with facilitating efficient data collection, we conducted group walks through the neighbourhoods. We consider that, on the whole, walking with others compared to walking as singletons is most likely to have attenuated levels of in situ anticipation of threat, perhaps more profoundly in neighbourhood 2 compared to neighbourhood 1. However, we consider that walking as a group instead of individually would perhaps have less direct effect on the sense of trustworthiness of the residents. Future research should explore how walking alone affects these judgements. It is also true that stopping to record judgements at places across the walk is likely to have augmented feelings of threat because of heightened self-consciousness. However, the walker may have felt a little less exposed doing this in a group compared to by themselves.

As our cities are increasingly 24 hour experiences, in situ appraisals of place could be collected to reflect this. For both practical and methodological reasons all of our group walks took place at the same time of day. Night time is most likely to show dramatic changes to in situ responses provided within both deprived and less deprived neighbourhoods with different potential threats felt across different areas within neighbourhoods. The salient features and sentiments reflected in them are also likely to be very altered at different times of the day/night.

Finally, it is relevant to acknowledge that even the less deprived neighbourhood in this study has pockets of severe deprivation within it and, typical of North West England, the area has an Index of Multiple Deprivation higher than the UK average. Thus, although these neighbourhoods contrasted greatly in terms of local level deprivation, neither can be considered average nor doing well. In this respect it is interesting to reflect that sense of threat could not be statistically modelled in neighbourhood 1 suggesting either that different dependent variables should have been considered or that levels of threat were too low to model accurately, a suggestion that is supported by the data presented in Table 2.

### Study implications

With the level of diagnosed mental health conditions continuing to rise in the UK, a country that is both intensively urban and supremely unequal, we believe that the small but growing body of evidence on urban psychology within which this study fits, has practical implications for how further increases in urban mental distress may be prevented. We also recommend that this type of mobile methodology, frequently used in the discipline of human geography, is adopted by the urban planning and regeneration professions as part of a wholly more inclusive approach to place-making that should also include joint decision-making and co-design [40].

The findings of this study provide the basis for more developed thinking about how our places affect us psychologically. It is notable that within the variables considered here no correlates of in situ threat in neighbourhood 1 were identified. This finding, along with the clue in this data that personal resilience may protect against feelings of threat in the more deprived neighbourhood, may indicate the existence of a critical ‘tipping point’. Perhaps when place characteristics highlighting harshness or impoverishment reach a certain level they become intolerable and begin to act negatively and predictably on the psychology of those who visit or dwell in them [33]. If this is so, it becomes a priority for public health and local governance to ensure that our urban neighbourhoods do not fall below this standard.

## Supporting information

S1 File The data upon which the analysis of this study was carried out. (SAV)
